# Rear impact responses of 50th percentile female and male dummies in a seat with different whiplash injury risk reduction for women and men

**DOI:** 10.3389/fbioe.2026.1837076

**Published:** 2026-08-21

**Authors:** Anna Carlsson, Anders Kullgren, Mats Y. Svensson

**Affiliations:** 1 Chalmers Industriteknik, Gothenburg, Sweden; 2 Vehicle Safety Division, Chalmers University of Technology, Gothenburg, Sweden; 3 Folksam Insurance Group, Stockholm, Sweden

**Keywords:** crash test dummy, females, injury risk, NIC protraction, rear impact, sled testing, WAD, whiplash

## Abstract

The aim of this study was to investigate the difference in rear impact dynamic response between a male and a female crash test dummy, in Saab 9–3 seats fitted with the Saab Active Head Restraint (SAHR) whiplash injury mitigation system. According to real world injury data, this SAHR system offers a greater risk reduction for the male population in comparison to the female. Identifying response differences between the male and female dummies, could explain the risk differences seen in real world crashes. Six sled tests were performed in Saab 9–3 seats (model year 1998–2002) in accordance with the European New Car Assessment Programme (Euro NCAP) whiplash test procedure (medium pulse, Δv 16 km/h, max acceleration 10 g). Two different low speed rear impact crash test dummies were used for the tests; the 50th percentile female prototype BioRID P50F_V2 and the 50th percentile male BioRID II. Both dummies exhibited early Head Restraint (HR) contact times which resulted in Neck Injury Criterion (NIC) values well below the recommended limit of 15 m^2^/s^2^. On the other hand, the NIC_protraction_ values were almost two times greater for the female dummy in comparison to the male. In the Saab 9–3 seat, the SAHR system may cause a forward push to the head for average sized females, resulting in protraction of the neck rather than retraction. Current whiplash protection testing is performed with a 50th percentile male dummy. However, the results of this study show that in order to explain the sex differences found in real world data, it is necessary to use sex specific sizes of dummies developed for rear impact testing and include injury criteria to cover also flexion and protraction motion in the dynamic response. To cover the majority of the adult population, we recommend using the 50th percentile female and male dummies.

## 1 Introduction

Whiplash injuries, often denoted Whiplash Associated Disorder (WAD), still account for most injuries resulting in long-term consequences in car crashes, despite new seat designs aimed at lowering the risk of whiplash injury and Automated Emergency Braking (AEB) aimed at reducing the number of rear impacts or the impact speed (Kullgren et al., 2020). The incidence and risk of whiplash injury vary between males and females. According to a review by Carlsson (2012), accident data have shown that females typically are exposed to twice the risk of sustaining whiplash injuries than males, even in similar crash conditions. On average, cars equipped with advanced whiplash protection systems pose a ∼40% lower risk of whiplash injury resulting in permanent medical impairment compared to cars fitted with standard seats (Kullgren et al., 2013). The injury reducing effect seems to vary between seat concepts aimed at reducing whiplash injury risk (Kullgren et al., 2010; 2013), but also between occupants of different ages and sexes (Kullgren et al., 2013; 2020).

Since 1997 several seat concepts aimed at reducing the risk of whiplash injury have been introduced. In 1997, Saab introduced the Saab Active Head Restraint (SAHR) system (Wiklund and Larsson, 1998). The following year, Volvo introduced their Whiplash Protection System (WhiPS) (Lundell et al., 1998) and Toyota their Whiplash Injury Lessening (WIL) system (Sekizuka, 1998). The injury reducing effect of the SAHR system has been evaluated by Viano and Olsén (2001), Farmer et al. (2003), Kullgren and Krafft (2010) and Kullgren et al. (2013), which ranges from 33% to 75% in these studies. However, the SAHR concept seems to have a significant effect for men (more than 70%) and limited or no effect for females, while both the WhiPS and the WIL concepts appear equal in reducing the risk for males and females (Kullgren and Krafft, 2010; Kullgren et al., 2013).

It is important to understand the reason for such differences in the SAHR system effectiveness for females and males. The SAHR reactive head restraint is connected to a pressure plate in the seatback by means of a spring-loaded link mechanism. When the seat pushes the occupant forward with more force than the spring can resist, the plate yields rearward into the seatback. This forces the head restraint (HR) to move upward and forward, thus closing the gap to the head before the relative motion between the head and the torso becomes significant (Wiklund and Larsson, 1998). However, ideal support of the head requires balance between a number of parameters. For example, the support of the head is significantly influenced by the vertical position of the height adjustable HR. Furthermore, occupant seated height as well as upper body mass and shape are important parameters for the SAHR activation. Carlsson et al. (2017) investigated the seated positions of drivers of Saab 9–3 vehicles in stationary conditions. The study showed that the majority adjusts the HR to a high position; 89% in any of the three uppermost positions and 59% in the top position. Furthermore, Carlsson et al. (2017) found that the horizontal backset tended to be shorter for females (27 mm) compared to males (44 mm). The backset for most of the Saab 9–3 drivers was within the recommended limits (Stemper et al., 2006); the backset was below 60 mm for 88% of the females and 69% of the males. Thus, based exclusively on the static measurements, incorrect adjustment of the HR could not explain the effectiveness differences of the SAHR system found between females and males. Instead, the answer may be found in the dynamic interaction between the occupant and the HR/seatback during a rear impact. Based on this assumption, Carlsson et al. (2017) suggested that the combination of a (re)active HR and a shorter backset, may produce a forward push to the head in females, resulting in protraction of the neck (neck forward S-shape) rather than retraction (neck rearward S-shape).

This would correspond to a whiplash injury mechanism otherwise seen in frontal collisions. Boström et al. (2000b) introduced an extended definition of NIC, NIC_generic_, which was applicable in both retraction and protraction loading of the neck:

$${\text{NIC}}_{\text{generic}}=0.2\times a_{\text{rel}}+v_{\text{rel}}\times \left|v_{\text{rel}}\right|$$

where a_rel_/v_rel_ are the relative T1-to-head centre of gravity x-acceleration/x-velocity according to SAE J211 conventions.

For the protraction phase in frontal collisions, they proposed the criterion NIC_protraction,_ which focuses on the negative peak of the NIC_generic_ curve and was derived from the injury mechanism suggested by Svensson et al. (2000), in which pressure transients in the spinal canal induced nerve root ganglion damage, both in retraction and protraction motion of the neck. Muser et al. (2002) explicitly suggested that the minimum of the NIC_generic_ curve, potentially could be used to indicate whiplash injury risk during the second phase of a rear-end collision as well as for frontal collisions.

Volunteer rear impact tests show that the dynamic response of 50th percentile females is clearly different from the 50th percentile male response (Linder et al., 2008; Carlsson et al., 2011; 2012; Carlsson, 2012). Since the 50th percentile male BioRID II has been validated based on tests with male volunteers, current whiplash protection seat concepts are designed without any consideration of female properties. Carlsson et al. (2021b) presented a first 50th percentile female prototype rear impact crash test dummy, BioRID P50F_V1. Schmitt et al. (2012) performed a series of rear impact sled tests with the male BioRID II and the female BioRID P50F_V1 in four standard vehicle seats. These tests clearly showed different results between the male and female dummies. Recently, an improved version of the female prototype dummy, called BioRID P50F_V2, was presented by Carlsson et al. (2026). This prototype dummy was evaluated based on volunteer data by Carlsson et al. (2021a).

The aim of this study was to perform a series of low-speed rear impact sled tests to identify differences in dynamic response between the female and male dummies in a Saab 9–3 (model year 1998–2002) seat, fitted with SAHR, to find a possible explanation for the difference in effectiveness between males and females shown in real world crashes.

## 2 Materials and methods

**FIGURE 1:**
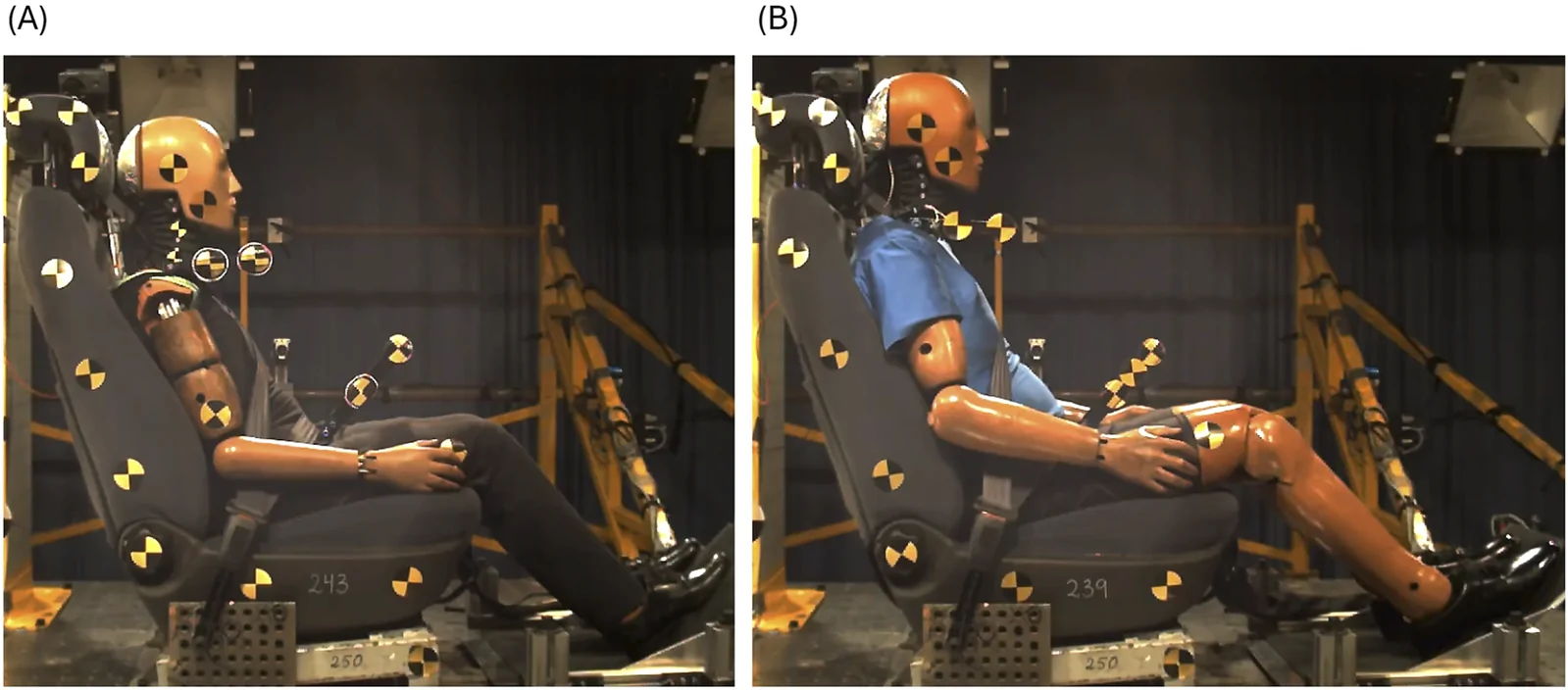
Test setup for **(A)** the prototype BioRID P50F_V2 and **(B)** the male BioRID II, both seated in the Saab 9–3 seat with the HR in the top position. Photos: Autoliv Sweden.

A series of six sled tests was performed in Saab 9–3 seats in accordance with the European New Car Assessment Programme (Euro NCAP) whiplash test procedure (medium pulse, Δv 16 km/h, max acceleration 10 g) (Euro NCAP, 2010). Two different low speed rear impact crash test dummies were used for the tests; the 50th percentile female prototype BioRID P50F_V2 (Figure 1A; Carlsson et al., 2026) and the 50th percentile male BioRID II (Figure 1B).

Six manually adjustable front passenger seats (fabric-covered) originating from the five-door Saab 9–3 (model years 1998–2002) were chosen for this study. The front seats of this car model are equipped with the SAHR Generation I whiplash protection system (Wiklund and Larsson, 1998). Neither of the six seats were height adjustable. The seats were replaced between tests, i.e., each seat was only tested once to ensure that pre-impact damage would not influence the response. The H-point and the seatback angle was determined using the SAE J826 manikin (Serial No. 365-HR, Automotive Accessories Ltd, Southampton, England).

The tests were performed at three different HR positions, low, mid and top, in accordance with the test matrix (Table 1; Supplementary Table S1). Please note that Carlsson et al. (2017) found that only a minor part (11%) of the Saab 9–3 drivers had HR adjusted in positions lower than the mid.

**TABLE 1:** Test matrix.

Dummy	HR model	HR position
BioRID P50F_V2 (prototype)	New^a^	Top^c^
	New^a^	Mid^d^
	Old^b^	Low^e^
BioRID II	New^a^	Top^c^
	New^a^	Mid^d^
	Old^b^	Low^e^

^a^ Six height settings (new HR model)

^b^ Ten height settings (old HR model)

^c^ 6(6)

^d^ 4(6)

^e^ 1(10).

The BioRID P50F_V2 was equipped with uniaxial accelerators in the head (x/y/z; Endevco 7264B/7264C-100 g), 1st thoracic vertebra (T1) (x/z; left/right side; Endevco 7264B-100 g), and a triaxial accelerometer in the pelvis (x/y/z; Endevco 7267A-200 g). The BioRID II was equipped with uniaxial accelerometers in the head (x/y/z) (Endevco 7264B-100 g), T1 (x/z; left/right) (Endevco 7264B-100 g), and a triaxial accelerometer in the pelvis (Endevco 7267A-200 g). A linear accelerometer (Entran EGCS-D1CM-100 g) was attached to the sled.

The accelerometer coordinate systems were defined according to the SAE J211 standards (orthogonal right-handed). The data acquisition unit Kayser-Trede MiniDau registered the sensor data at a sampling rate of 20 kHz, and the data were anti-alias filtered at 2.9 kHz (6 pole Bessel-filter). The accelerations were filtered according to the channel frequency class (CFC) of the SAE J211/1, (2007) standard (sled: CFC 60, head: CFC 1000, T1: CFC 60).

Film targets were secured on the BioRID P50F_V2, the BioRID II, and on the seats prior to the tests (Figure 1). The same seat configuration and dummy positioning were used for the BioRID P50F_V2 as for the BioRID II, based on the Euro NCAP test procedure (Euro NCAP, 2010). The pelvis angle of the BioRID P50F_V2 was adjusted in accordance with the pelvis angle of the BioRID II (target range: 26.5° ± 1°; Euro NCAP: 26.5° ± 2.5°). The head was positioned horizontally with respect to a spirit-level attached to the top of the head. The T1 angle of the BioRID P50F_V2 was noted (measured on the T1 bracket mounted 32° relative to the back of the T1 vertebra). Pre-test, the following parameters were registered.

➢ Backset: the shortest horizontal head-to-HR distance. This distance was measured using a caliper.
➢ Offset: the vertical distance between the top of the head and the top of the HR.

The tests were monitored by three high-speed digital video cameras (Phantom V6.2; 512 × 512 pixels; 1,000 frames per second; exposure time 150 ms); one overview and one detail view from the right side, and one overview from the left side. The cameras were attached to the sled, perpendicular to the direction of travel, approximately 2.2 m from the dummy head. The digital video camera data was digitised and depth scaled using Tema 3.12 software with an accuracy of ∼1 mm. None of the displacement data were filtered. Displacements of the head and T1 were derived from targets 1) and 3), respectively (Supplementary Figure S1). Angular displacements of the head and T1 were derived from targets 1) and 2) as well as 4) and 5). Seatback angular displacements of the Saab 9–3 seats were derived from targets 6) and 7). The displacement data was set to zero at the time of impact (T = 0) and was expressed in a sled fixed coordinate system.

**FIGURE 2:**
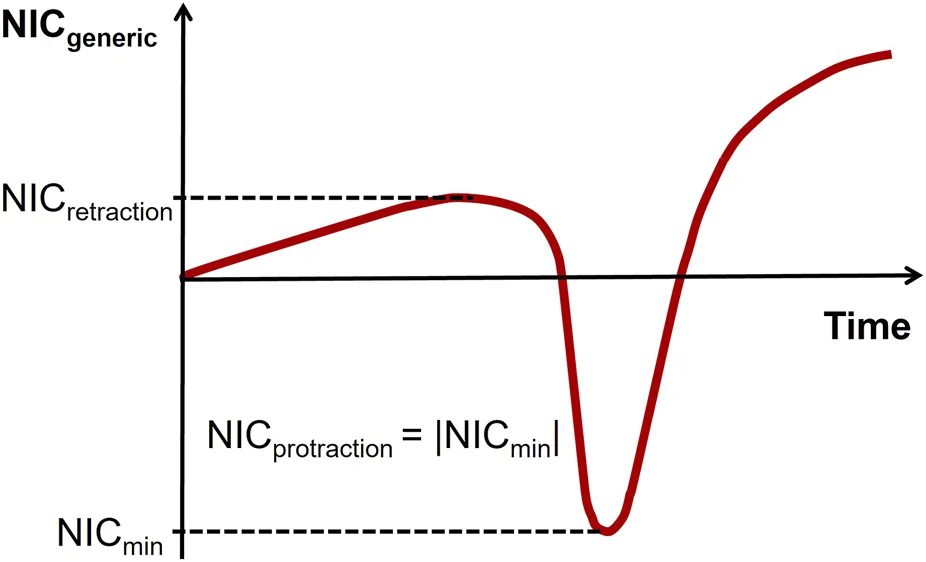
Illustration of a typical time history of a NIC curve in a rear impact test (based on Eichberger et al., 2000) and how the maximum NIC_retraction_ value is identified, together with the corresponding definition of the extreme value of NIC_protraction_.

The peak values of the head and spine x-accelerations and the x- and angular displacements, and their occurrence in time, were determined for each test. The head-to-HR contact time was recorded. Here we introduced the new term NIC_retraction_, defined as maximum NIC_generic_, in the time window of the initial retraction phase. We also used the NIC_protraction_, defined as the absolute value of the minimum of NIC_generic_, in the phase immediately after retraction. The two initial phases corresponding to NIC_retraction_ and NIC_protraction_, are directly linked to the proposed injury mechanism related to cervical nerve root ganglion injuries, induced by pressure transients in the spinal canal (Svensson et al., 2000). It was originally proposed that the NIC would be calculated at the initial event of maximal retraction (Boström et al., 2000c), which is how NIC_retraction_ is currently used. In practice, the NIC_protraction_ is intended for the initial event of maximum protraction, and Muser et al. (2002) have suggested that the minimum of the NIC_generic_ curve, could be used to indicate whiplash injury risk during the second phase of a rear-end collision. Figure 2 is based on a figure by Eichberger et al. (2000) that illustrates the typical time history of a NIC curve in a rear impact test and how the maximum NIC_retraction_ value is identified. In Figure 2 we also illustrate the corresponding definition of the extreme value of NIC_protraction_.

## 3 Results

The three positions of the HR (low, mid, top) strongly affected the position of the head relative to the HR of the two dummies (Supplementary Figure S2). Consequently, the vertical offset varied greatly, ranging from -28–73 mm for the BioRID P50F_V2 and 49–151 mm for the male BioRID II (Table 2). Overall, the BioRID P50F_V2 had a better vertical offset geometry rating (acceptable, good, good) (Zuby and Lund, 2010) than the BioRID II (poor, acceptable, good) (Table 2). In the mid and top HR positions, the horizontal backset was slightly greater for the BioRID P50F_V2 (30 mm) in comparison to the BioRID II (25 mm) (Table 2). In the low HR position, the female dummy had somewhat greater backset (44 mm), however, measuring for the male dummy was not possible due to the top of the HR being placed below the head centre of gravity. Thus, the BioRID P50F_V2 had a better horizontal backset geometry rating (good, good, good) than the BioRID II (poor, good, good), (Table 2).

**TABLE 2:** Summary of results from the tests comprising BioRID P50F_V2 and BioRID II in the Saab 9–3 seats at three different HR positions (Low, Mid, Top).

Variable	BioRID P50F_V2						BioRID II					
	Low		Mid		Top		Low		Mid		Top	
	Peak	Time	Peak	Time	Peak	Time	Peak	Time	Peak	Time	Peak	Time
Head restraint	[mm]	[ms]	[mm]	[ms]	[mm]	[ms]	[mm]	[ms]	[mm]	[ms]	[mm]	[ms]
- Backset	44	​	30	​	30	​	Not applicable		25	​	25	​
- Backset rating^d^	Good		Good		Good		Poor		Good		Good	
- Offset	73	​	4	​	−28	​	151	​	73	​	49	​
- Offset rating^d^	Acceptable		Good		Good		Poor		Acceptable		Good	
- Contact	​	49	​	46	​	46	​	86	​	47	​	47
x-displacement^c^	[m]	[ms]	[m]	[ms]	[m]	[ms]	[m]	[ms]	[m]	[ms]	[m]	[ms]
- Head	−0.16	95	−0.14	84	−0.14	85	−0.28	114	−0.21	106	−0.19	100
- T1	−0.15	98	−0.13	89	−0.14	91	−0.17	107	−0.18	102	−0.16	99
x-acceleration	[m/s^2^]	[ms]	[m/s^2^]	[ms]	[m/s^2^]	[ms]	[m/s^2^]	[ms]	[m/s^2^]	[ms]	[m/s^2^]	[ms]
- Head^a^	159	60	149	61	139	60	179	94	79	58	86	57
- Head^b^	171	88	127	91	126	77	228	112	150	106	143	101
- T1	121	70	118	72	123	86	95	114	134	83	103	83
- Pelvis	122	76	129	79	128	75	110	78	107	84	102	73
Ang. displacement	[°]	[ms]	[°]	[ms]	[°]	[ms]	[°]	[ms]	[°]	[ms]	[°]	[ms]
- Head	11.2	96	7.2	85	6.9	88	33.2	120	14.1	107	12.8	106
- T1	10.7	97	6.2	94	6.7	90	26.4	120	14.9	112	12.6	107
- Seatback	7.3	81	5.7	83	7.1	88	10.1	99	12.3	97	10.6	93
NIC	[m^2^/s^2^]	[ms]	[m^2^/s^2^]	[ms]	[m^2^/s^2^]	[ms]	[m^2^/s^2^]	[ms]	[m^2^/s^2^]	[ms]	[m^2^/s^2^]	[ms]
- NIC_retraction_	5.6	49	4.8	46	4.2	46	12.3	50	4.3	47	3.5	48
- NIC_protraction_	19.3	60	13.2	61	13.4	59	Not applicable		6.4	54	7.3	54

^a^ First peak

^b^ Second peak

^c^ Relative to the sled

^d^ Zuby and Lund (2010).

**FIGURE 3:**
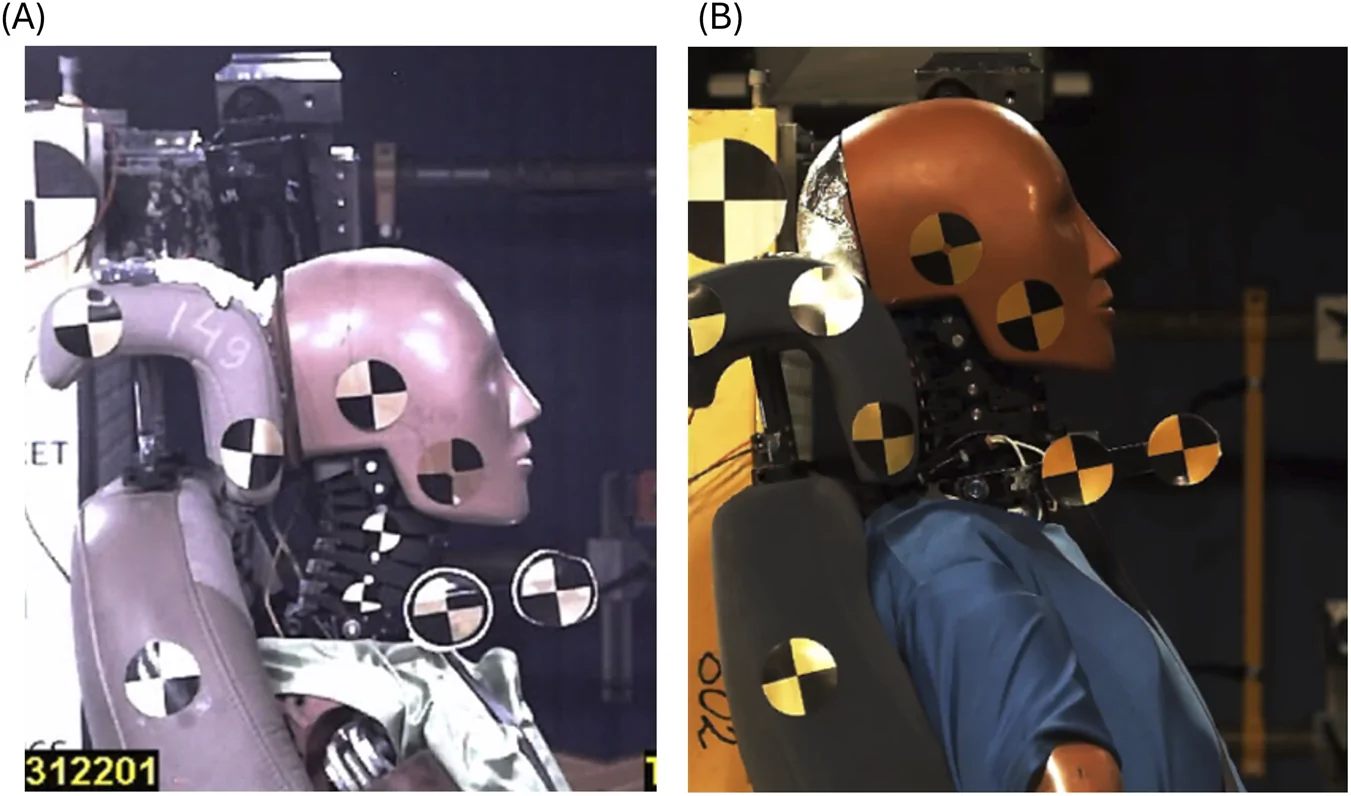
The female dummy **(A)**, sinks deeper into the seatback compared to the male dummy **(B)**. These two video frames correspond to the time of maximum rearward T1 displacement in the HR mid position tests. In contrast to the male **(B)** the neck of the female **(A)** is in pronounced protraction.

**FIGURE 4:**
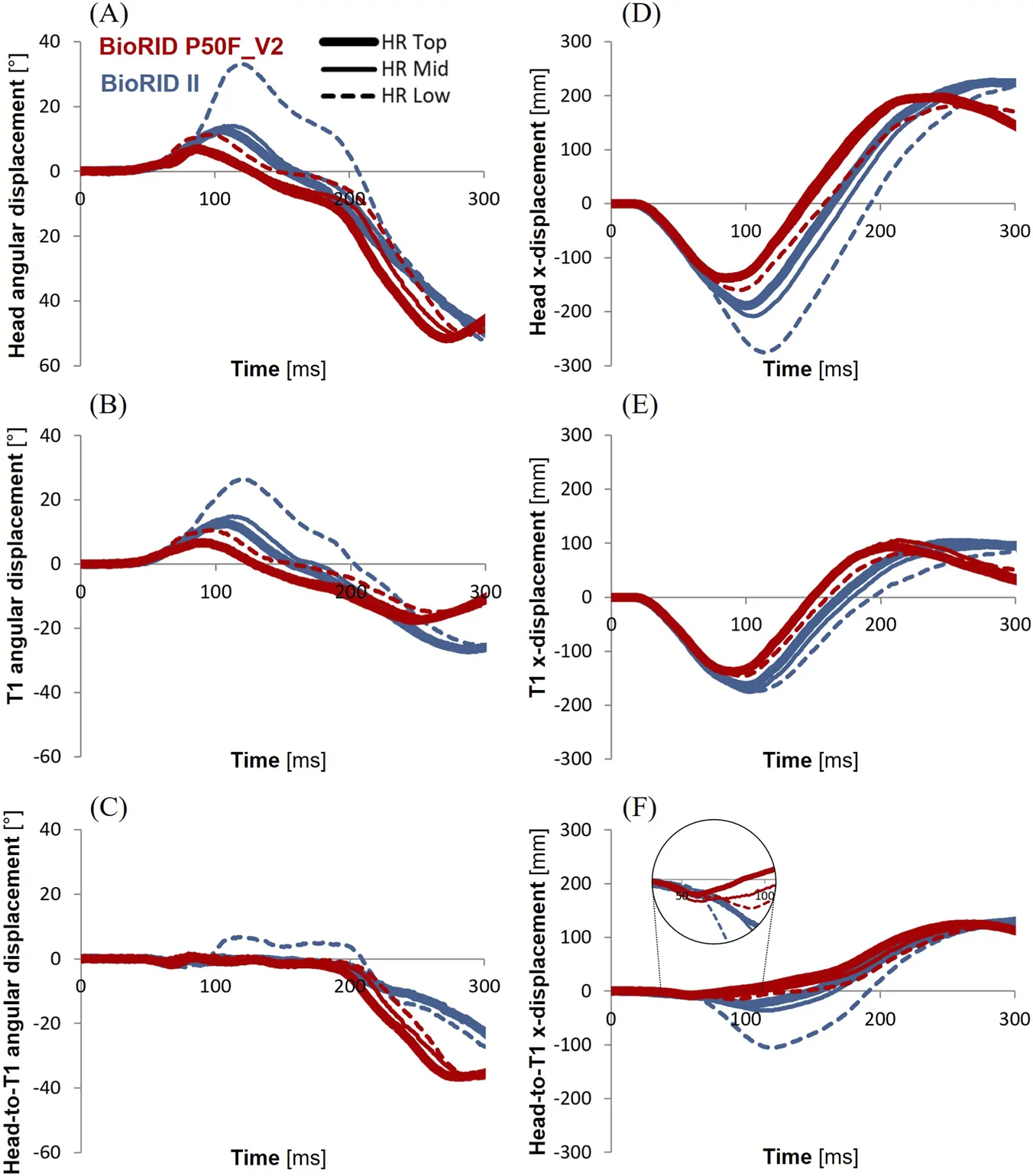
Angular displacement of the **(A)** head, **(B)** T1, and **(C)** head-to-T1, and x-displacement (relative to the sled) of the **(D)** head, **(E)** T1 and **(F)** head-to-T1, for the BioRID P50F_V2 (red) and BioRID II (blue) at three different HR positions; top (solid thick line), mid (solid thin line) and low (dotted line). The head-to-T1 x-displacement is very similar for the BioRID P50F_V2 and BioRID II up to approximately 60 ms, however at this time point the BioRID P50F_V2 head motion suddenly accelerated forward relative to T1. This abrupt change is shown enlarged and coincides with the BioRID P50F_V2 head x-acceleration peak at 60 ms (Figure 5A).

The motion of the head/neck was much influenced by the position of the HR in relation to the head (Supplementary Figures S3, S4). In all HR positions, the female dummy head underwent a very small retraction motion before it was pushed forward by the HR, resulting in a protraction of the neck (Figure 3A) rather than retraction. This was not seen in the male dummy (Figure 3B). Furthermore, in the low HR position, the head of the BioRID II did not receive enough support, resulting in a considerably greater head rearward angular displacement (33.2°) in comparison to the mid (14.1°) and top (12.8°) HR positions (Figure 4A; Table 2). The BioRID P50F_V2 had less rearward head angular displacements in all 3 HR positions, low (11.2°), mid (7.2°) and top (6.9°). Similarly, the T1 rearward angular displacements of the BioRID P50F_V2 were less in all 3 HR positions, low (10.7°), mid (6.2°) and top (6.7°), compared to the male dummy (26.4°, 14.9° and 12.6°, respectively) (Figure 4B; Table 2). Interestingly, the head-to-T1 angular displacement curves remained close to zero until ∼200 ms (Figure 4C), which shows that the head moved in an almost pure retraction/protraction motion during this time frame.

**FIGURE 5:**
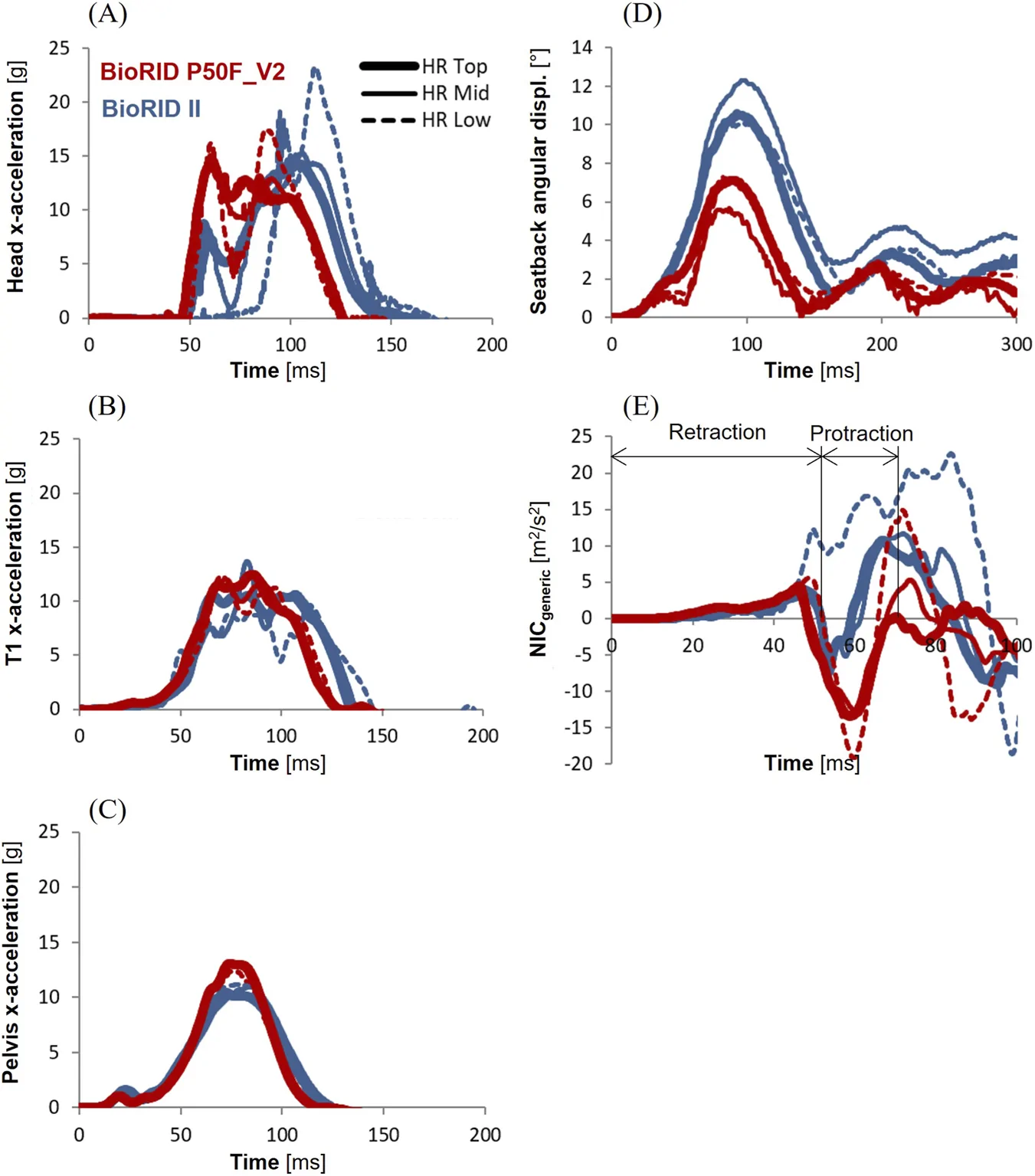
x-displacement of the **(A)** head, **(B)** T1, and **(C)** pelvis, **(D)** angular displacement of the seatback **(E)** NIC_generic_, for the BioRID P50F_V2 (red) and BioRID II (blue) at three different HR positions; top (solid thick line), mid (solid thin line) and low (dotted line). The NIC_generic_ criterion **(E)** applies both to the retraction and the protraction phases of the neck motion. The NIC_retraction_ concerns the retraction phase which corresponds to the initial positive section of the NIC_generic_ curve (0–50 ms). The NIC_protraction_ corresponds to the absolute value of the first negative NIC_generic_ peak **(E)** which occurs in the neck protraction phase (40–60 ms).

The average seatback rearward angular displacement was 39% less for the BioRID P50F_V2 (6.7°) in comparison to the BioRID II (11.0°) (Figure 5D; Table 2). Therefore, the peak rearward x-displacements of the head and T1 were less and earlier for the BioRID P50F_V2 compared to the BioRID II (Figures 4D,E; Table 2). At the mid/top HR positions, the peak rearward head x-displacements were 32%/28% less and 21%/15% earlier for the female dummy, while the T1 was 23%/16% less and 13%/8% earlier. The head-to-T1 x-displacement (Figure 4F) is very similar for the BioRID P50F_V2 and BioRID II, until approximately 60 ms when the BioRID P50F_V2 head motion turns sharply forward relative to T1. This sharp change is magnified in Figure 4F and coincides with the BioRID P50F_V2 head x-acceleration peak at 60 ms (Figure 5A).

Also, the female dummy had on average 13%/19% shorter head-to-HR contact time for the mid/top HR positions. Consequently, the rebound motion was faster for the female dummy, with an earlier return to the initial position (=0 cm) of the head and T1 (Figures 4D,E). At the mid/top HR positions, the head of the BioRID P50F_V2 returned to the initial positions 16%/14% earlier than the head of the BioRID II; similar results were recorded for the T1, 16%/12% earlier.

The NIC_generic_ (Figure 5E) criterion applies both to the retraction and the protraction phases of the neck motion. NIC_retraction_ concerns the retraction phase which corresponds to the initial positive section of the NIC_generic_ curve. At 50 ms, the NIC_generic_ curves have passed the first peak for the mid/top HR positions and are in a negative slope (Figure 5E). Therefore, 50 ms was set as the breaking point between the retraction phase and the protraction phase. In the mid/top HR positions, the NIC_retraction_ values were similar for the BioRID P50F_V2 and BioRID II (on average 4.5 m^2^/s^2^ and 3.9 m^2^/s^2^, respectively) (Figure 5E; Table 2). For both the female and male dummy, the NIC_retraction_ values tended to be greater in the low HR position (5.6 m^2^/s^2^ and 12.3 m^2^/s^2^, respectively) in comparison to the mid (4.8 m^2^/s^2^ and 4.3 m^2^/s^2^, respectively) and top (4.2 m^2^/s^2^ and 3.5 m^2^/s^2^, respectively). The NIC_protraction_ value corresponds to the absolute value of the first negative NIC_generic_ peak (Figure 5E), which occurs in the neck protraction phase following the time point 50 ms. In the present study, we only took the first negative NIC_protraction_ peak into consideration, based on the same assumption as for the NIC_retraction_. As all NIC_protraction_ peaks for the mid and top HR positions occur in the interval 50–70 ms, only values within this interval were included in the analysis. The NIC_protraction_ values had much greater magnitudes for the female dummy in all 3 HR positions (low: 19.3 m^2^/s^2^, mid: 13.2 m^2^/s^2^, top: 13.4 m^2^/s^2^) in comparison to the male dummy (low: not applicable, mid: 6.4 m^2^/s^2^, top: 7.3 m^2^/s^2^).

The shape of the head x-acceleration pulses differed between the female and the male dummies, especially in the low HR position where the male dummy had a late head-to-HR contact due to the poor HR geometry (Figure 5A; Table 2). The head x-acceleration tended to have two peaks in all tests apart from the test with poor HR geometry. In the mid and top HR positions, the BioRID P50F_V2 had a greater 1st peak and a lesser 2nd peak, while the opposite was found for the BioRID II. This resulted in the 1st peak being on average 74% greater and the second peak 14% lesser for the female in comparison to the male dummy. In the low HR position, both dummy sizes had a greater 2nd peak and a lesser 1st peak, (Figure 5A; Table 2). The peak T1 x-accelerations were similar for the male and female dummies (Figure 5B; Table 2), while for the pelvis they were on average 23% greater for the BioRID P50F_V2 (Figure 5C; Table 2).

**FIGURE 6:**
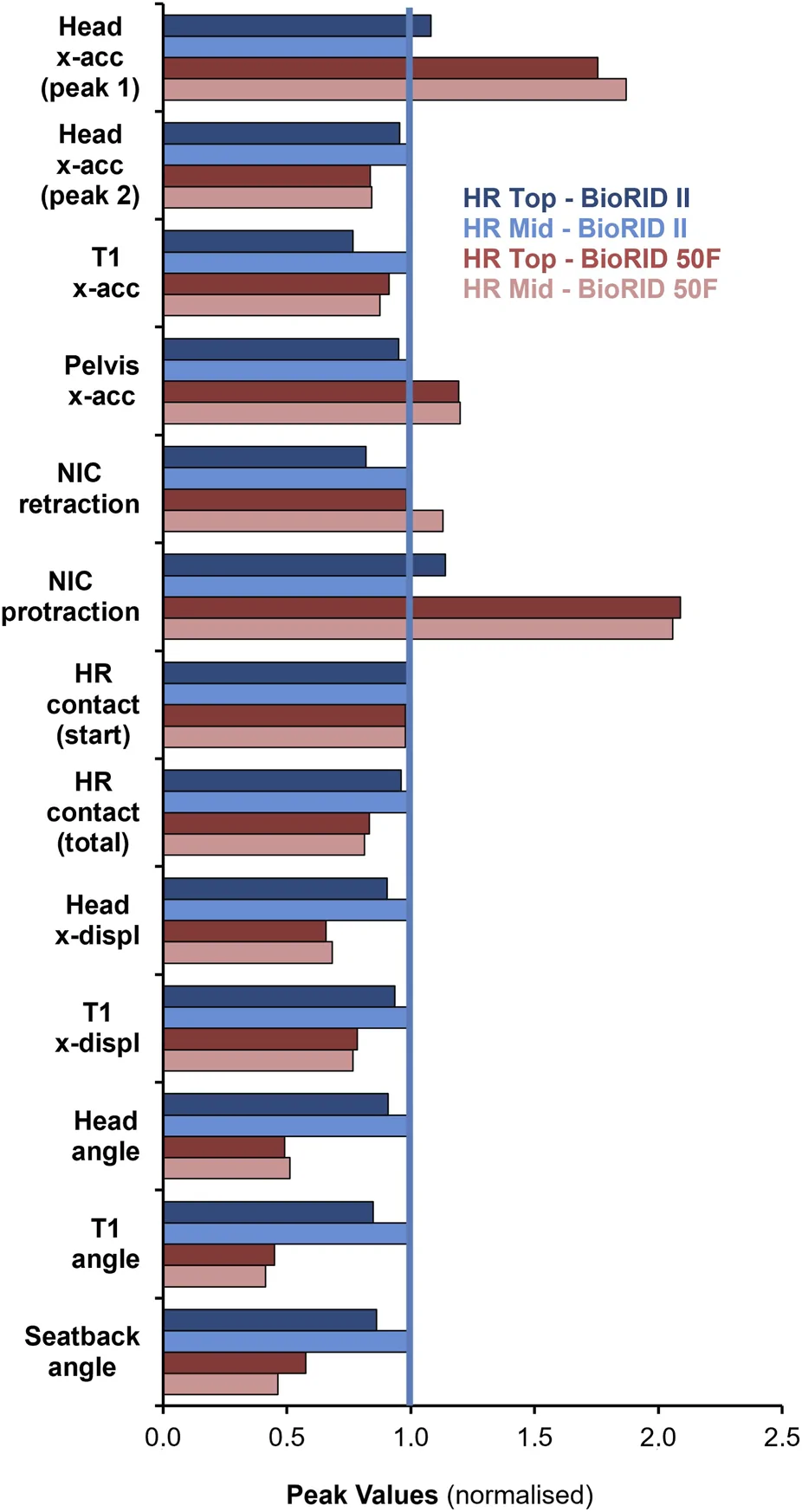
Overview of peak values for the mid and top HR positions. Each peak is normalised with regard to the BioRID II in the mid HR position (=1). Please note that NIC_protraction_ corresponds to the negative NIC sequences that peak in the interval 50–70 ms in the present study (Figure 4E).

An overview of the normalised peak values for the mid and top HR positions is presented in Figure 6. Carlsson et al. (2017) found 89% of Saab 9–3 drivers had adjusted the HR to any of the three uppermost positions, corresponding to the mid-to-top HR positions, and as many as 59% in the top position. Thus, Figure 6 shows the peak values for the most relevant HR positions.

## 4 Discussion

Real-world data clearly shows that females are at greater risk of sustaining whiplash injuries compared to males (Carlsson, 2012; Kullgren et al., 2020). Although several whiplash preventive concepts have been developed and tested, generally the difference in risk remains. Several studies have shown that many whiplash protection systems offer different levels of risk reduction for females and males. The SAHR is of the Reactive Head Restraint (RHR) type, and according to real world injury data these systems offer a greater risk reduction for the male population in comparison to the female (Kullgren and Krafft, 2010; Kullgren et al., 2013).

The aim of this study was to perform a series of low-speed rear impact sled tests to investigate the differences in dynamic response between the female and male dummies, in a Saab 9–3 (model year 1998–2002) driver seat fitted with an RHR, which has shown a significant difference in real-world performance between males and females. For the Saab 9–3, the male risk of injury was reduced by 70% compared to the preceding Saab model without the SAHR system, but there was an insignificant reduction in risk for females (Kullgren et al., 2013).

The seat performance was evaluated using the Euro NCAP mid severity pulse at 16 km/h with a triangular shape and a peak acceleration of 10 g (van Ratingen et al., 2009). This crash pulse was generated in 2003 by the International Insurance Whiplash Prevention Group (IIWPG), designed specifically to assess the performance of seats and head restraints (Edwards et al., 2005). The test speed of 16 km/h represents a change of velocity close to the average for car occupants sustaining whiplash symptoms for more than 1 month (Kullgren and Krafft, 2008). In addition to the BioRID II, the setup also included the prototype dummy BioRID P50F_V2 which has been shown to closely resemble the female volunteer response corridors (Carlsson et al., 2026). Thus, the BioRID P50F_V2 provides a unique opportunity to compare male and female responses in rear impact test.

At the time of planning the tests herein, a bio-fidelic alternative to the BioRID P50F_V2 was not available to represent the average female in rear impact testing. Wang et al. (2025) published modelling results from the FE-model of the average sized female rear impact dummy EvaRID (v1.5.1), in which they also stated that “no physical tests were conducted because the EvaRID physical dummy was not built yet”. Another potential alternative for future rear impact testing would be the two versions of the Seat Evaluation Tool (SET), the average male sized SET 50M and the average female sized SET 50F (Karemyr et al., 2022). The SETs were primarily developed to bridge the gap between physical testing and FE-simulation, as part of a tool chain for virtual testing. In this case, the two SETs have been used to assess the dynamic response of a car seat, while the corresponding FE-SET (Alvarez and Brolin, 2024) then represents the physical SETs in the FE-environment, in which the FE-model of the same car seat was finally validated.

The results showed that the female dummy had 39% less peak seatback rearward angular displacement in comparison to the male dummy, which was expected due to its lesser mass and lower centre of gravity. Consequently, the upper body of the female dummy had a shorter contact duration with the seatback, resulting in greater head and T1 x-accelerations and earlier rebound. In the mid and top HR positions, corresponding to the most common settings in real life, the female head and T1 rearward angular and x-displacements were substantially less than those of the male (Table 2; Figure 6).

We decided to introduce the injury criterion NIC_retraction_ for the early neck retraction phase (Figure 5E), rather than the commonly used NIC_max_, as the NIC criterion was originally intended for calculation at the event of maximal retraction (Boström et al., 2000c). In the following protraction phase (50–70 ms) we used the NIC_protraction_ (Boström et al., 2000b). NIC_retraction_ and NIC_protraction_ represent the two initial phases in the NIC_generic_ curve (Figure 2), and they are linked more directly to the proposed injury mechanism related to the cervical nerve root ganglia, induced by a pressure pulse in the spinal canal (Svensson et al., 2000). Except for the male dummy in the low HR position, the NIC_retraction_ values were well below the recommended limit of 15 m^2^/s^2^ (Boström et al., 2000c) (Table 2).

The NIC_protraction_ values were however on average almost two times greater for the female dummy in comparison to the male (Table 2; Figure 6). This clearly demonstrates that the differences in injury risk between females and males in the seat tested may be explained by the protraction, as suggested by Carlsson et al. (2017), rather than the retraction. This is also reflected in Figure 3, showing that the HR pushes the head of the female dummy forward, resulting in a pronounced neck protraction. A corresponding motion was not observed in the male dummy. Interestingly, forward protraction motion of the cervical spine generated the same type of pressure transients and pressure induced ganglion injuries as in rearward retraction motion (Svensson et al., 2000). This is important as well as concerning, since a criterion aimed at measuring critical loads in protraction motion in rear impact testing has not been established to date. Furthermore, this issue may also be applicable to other potential injury mechanisms, as well as to whiplash injuries sustained in frontal collisions.

The head-to-HR contact occurred at similar times for the two dummies. This contrasts with earlier volunteer studies reporting that females on average have an earlier HR contact (Linder et al., 2008; Carlsson et al., 2012). The backset of the female dummy (30 mm) was somewhat greater than for the male dummy (25 mm). This contrasts with the previous study on Saab 9–3 drivers of Carlsson et al. (2017) which reported an average backset of 25 mm for mid-sized female test subjects, while the figure for mid-sized males was 41 mm. Thus, the backset of the female dummy was 5 mm greater, while the backset of the male dummy was 16 mm lesser, in comparison to the mid-sized test subjects. In addition, the SAHR moved towards the head in a forward/upward motion which can also influence the head-to-HR contact time. These backset differences in combination with the moving SAHR may explain why the timings were similar for the male and female dummies. The difference in backsets between the dummies and mid-sized volunteers may need to be further investigated in the future to gain a more complete understanding of the representativeness of the dummies in this aspect.

This study is unique in that both 50th percentile male and female dummies have been used when testing a specific vehicle seat, Saab 9–3, which has shown considerably greater risk of whiplash injury in real world rear impacts for females than for males. Carlsson et al. (2017) found that the HR in the Saab 9–3 is most often adjusted in any of the three top-most positions (89%), corresponding to the HR positions presented in Figure 6. Thus, the dynamic tests revealed that several important parameters differ between the female and male dummies, in particular the NIC_protraction_ and the head x-acceleration. This supports the assumption of Carlsson et al. (2017) that the combination of a (re)active HR and a shorter backset in females may produce a forward push to the head, resulting in protraction of the neck rather than retraction. The results clearly show that both male and female properties must be taken into consideration when testing whiplash protection systems. It was particularly important to use both male and female dummies when testing the SAHR system to assess potential differences based on sex, in dynamic response, which could explain the differences in injury risk shown in real-world crashes. Any future, fully developed female crash dummy, must be built from scratch with new components in order to precisely adapt the spine sections to the average female size, shape and properties. Thus, the introduction of a 50th percentile female rear impact dummy addresses a long-standing gap in vehicle safety by better representing the biomechanics and injury risk of the average female occupant. To be effective, its use must be accompanied by female-specific injury criteria, seating and HR adjustment procedures, and consideration of real-world seating preferences. Together, these measures will enable more accurate safety assessments and support the development of vehicle designs that offer equivalent protection for women and men alike.

The present study has also highlighted an interesting connection to concussion in rear impacts. Elkin et al. (2016) reported that the clinical presentations of whiplash injury and concussion show considerable overlap; both diagnoses are generally based on presenting signs and symptoms, in combination with a history of neck or head trauma. In a retrospective cohort study of concussions sustained in motor vehicle crashes, Tator et al. (2025) found that rear impact was the most common crash type, and that females were more frequently injured than males. Notably, females who sustained a concussion in rear impacts constituted the largest subgroup. Interestingly, the NIC_retraction_ and NIC_protraction_ criteria are based on a proposed injury mechanism involving pressure-induced dysfunction of the cervical nerve root ganglia (Svensson et al., 2000; Yao et al., 2018). This pressure-based mechanism was originally hypothesised by Aldman (1986), who described its potential to induce strain in neural tissues, in the cervical nerve root region as well as in the brain via the foramen magnum. The latter hypothesis was supported by experimental studies of concussion induced by pressure transients generated in a fluid percussion model in an animal model (Lindgren, 1983). The discovery of a potential link to concussion in rear impacts, further backs inclusion of an average sized female rear impact dummy, which would reinforce efforts to better understand why female occupants are at a higher risk of concussion in rear impacts.

To conclude, the female dummy showed a pronounced protraction in the test performed, which provides a plausible explanation for the differences found in real-world performance between females and males in the Saab 9–3. Thus, it is important to take female risk into account by also incorporating a female size dummy and corresponding test protocols when developing and evaluating future whiplash protection systems. Future whiplash protection assessments must consider additional injury criteria that also comprise head and neck protraction.

## Data Availability

The raw data supporting the conclusions of this article will be made available by the authors, without undue reservation.
