# Supplementary material for: Rear impact responses of 50th percentile female and male dummies in a seat with different whiplash injury risk reduction for women and men
Source: Front Bioeng Biotechnol. 2026 Aug 21;14:1837076. doi: 10.3389/fbioe.2026.1837076 (PMC13542634; doi:10.3389/fbioe.2026.1837076)
Supplement: Supplementary file 1 [file Supplementaryfile1.docx]

Supplementary Material

Depending on the model year, there are two different versions of the head restraint (HR) for this specific car model. The only difference between the restraints is that the older version has 10 distinct height settings instead of six. The six highest settings are identical for the two different models, whereas the older model has four extra lower positions (**Supplementary Table 1**).

| **Supplementary Table 1.** There are two different versions of HRs in the Saab 9-3, model years 1998–2002. The HR position equals the upmost position of the HR 100% for both models. The HR position equals the lowest position of the HR 0% in the new model, whereas -80% equals the lowest position in the old model. | | | | | |
| --- | --- | --- | --- | --- | --- |
|  | **HR Height** | | | **HR Setting** | |
|  | [%] | [mm] | | Old Model | New Model |
| Only  the old model | -80 | -44.8 | | 1(10) | - |
|  | -60 | -33.6 | | 2(10) | - |
|  | -40 | -22.4 | | 3(10) | - |
|  | -20 | -11.2 | | 4(10) | - |
| Same for  the old and  the new model | 0 | 0 | | 5(10) | 1(6) |
|  | 20 | 11.2 | | 6(10) | 2(6) |
|  | 40 | 22.4 | | 7(10) | 3(6) |
|  | 60 | 33.6 | | 8(10) | 4(6) |
|  | 80 | 44.8 | | 9(10) | 5(6) |
|  | 100 | 56.0 | | 10(10) | 6(6) |
| **Old Model** (10 height settings) | | | **New Model** (6 height settings) | | |
|  | | | | | |

**Supplementary Figure 1.** Video targets used to analyse the dynamic response of the BioRID P50F_V2 and the seatback.

|  | **BioRID P50F_V2** | **BioRID II** |
| --- | --- | --- |
| **HR Top** |  |  |
| **HR Mid** |  |  |
| **HR Low** |  |  |
| **Supplementary Figure 2.** Photos pre-test: Comparison BioRID P50F_V2 and BioRID II. | | |

| **HR Top – BioRID P50F_V2** (T=0) | **HR Top – BioRID P50F_V2** (max seatback angle) | |
| --- | --- | --- |
|  |  | |
| **HR Mid – BioRID P50F_V2** (T=0) | **HR Mid – BioRID P50F_V2** (max seatback angle) | |
|  |  | |
| **HR Low – BioRID P50F_V2** (T=0) | **HR Low – BioRID P50F_V2** (max seatback angle) | |
|  |  | |
| **Supplementary Figure 3.** Photos pre-test (T=0) and at the maximum seatback angle for the BioRID P50F_V2 at three different HR positions (low, mid, top). | |  |
|  |  | |

| **HR Top – BioRID II** (T=0) | **HR Top – BioRID II** (max seatback angle) | |
| --- | --- | --- |
|  |  | |
| **HR Mid – BioRID II** (T=0) | **HR Mid – BioRID II** (max seatback angle) | |
|  |  | |
| **HR Low – BioRID II** (T=0) | **HR Low – BioRID II** (max seatback angle) | |
|  |  | |
| **Supplementary Figure 4.** Photos pre-test (T=0) and at the maximum seatback angle for the BioRID II at three different HR positions (low, mid, top). | |  |
